# Advancements in pH-Responsive nanoparticles for osteoarthritis treatment: Opportunities and challenges

**DOI:** 10.3389/fbioe.2024.1426794

**Published:** 2024-07-01

**Authors:** Shuai Liao, Shicheng Jia, Yaohang Yue, Hui Zeng, Jianjin Lin, Peng Liu

**Affiliations:** ^1^ Department of Bone and Joint Surgery, Peking University Shenzhen Hospital, Shenzhen, China; ^2^ National and Local Joint Engineering Research Center of Orthopaedic Biomaterials, Peking University Shenzhen Hospital, Shenzhen, China; ^3^ Shenzhen University School of Medicine, Shenzhen, China; ^4^ Department of Sport Medicine, Peking University Shenzhen Hospital, Shenzhen, China; ^5^ Shenzhen Key Laboratory of Orthopaedic Diseases and Biomaterials Research, Shenzhen, China

**Keywords:** osteoarthritis, nanoparticles, pH responsive, intra-articular injection, drug delivery

## Abstract

Osteoarthritis (OA) is a degenerative disease linked to aging and obesity. The global aging population has led to an increasing number of OA patients, imposing a significant economic burden on society. Traditional drugs treatment methods often fail to achieve satisfactory outcomes. With the rapid advancement of nanomaterial delivery systems, numerous studies have focused on utilizing nanomaterials as carriers to achieve efficient OA treatment by effectively loading and delivering bioactive ingredients (e.g., drugs, nucleic acids) tailored to the unique pathological conditions, such as the weakly acidic microenvironment of synovial fluid in OA patients. This review highlights the latest advancements in the use of pH-responsive nanoparticles for OA treatment, emphasizing the principle of targeted drug delivery leveraging the acidic microenvironment of inflamed joints. It further discusses the composition, synthesis, response mechanism, target selection, application, and recent research findings of nanoparticles, while also addressing the challenges and future directions in this promising field.

## 1 Introduction

Osteoarthritis (OA) is one of the most common joint diseases worldwide, primarily caused by obesity and aging. By 2032, it is estimated that 29.5% of individuals over the age of 45 will have OA, with 15.7% affected by knee OA (Martel-Pelletier et al., 2016; Hunter and Bierma-Zeinstra, 2019). OA is a slowly progressing disease characterized by irreversible structural changes involving the entire joint, including articular cartilage, subchondral bone, ligaments, synovium, and joint capsule. Mechanical, inflammatory, and metabolic factors contribute to the gradual loss of joint function. (Brandt et al., 2006; Martel-Pelletier et al., 2016) The degeneration and destruction of articular cartilage are central to the pathophysiological changes of OA. The disease progression is marked by alterations in cartilage composition, leading to a gradual loss of its integrity. (Loeser et al., 2016).

As the disease advances, symptoms such as pain emerge. Hypertrophic chondrocytes increase their synthetic activity, producing matrix degradation products and pro-inflammatory mediators, including matrix metalloproteinases (MMPs) (Mehana et al., 2019), a disintegrin and metalloproteinase with thrombospondin motifs (ADAMTS) (Bondeson et al., 2008), Interleukin-1β(IL-1β), Interleukin-6(IL-6), and tumor necrosis factor (TNF) (Glyn-Jones et al., 2015). These factors promote the proliferation of adjacent synovial cells and inflammatory responses, with synovitis being a primary source of pain. Ultimately, OA leads to the loss of joint function, severely impacting patients’ quality of life.

Current treatment for osteoarthritis (OA) varies according to the disease’s severity. (Nelson et al., 2014; Bannuru et al., 2019; Gibbs et al., 2023) In early-stage OA, pain symptoms are not prominent, and joint range of motion is not significantly limited. Numerous guidelines recommend non-drug treatments as the first line of treatment, emphasizing patient health education, self-management, exercise, and weight loss. (Block et al., 2014; Nelson et al., 2014) When pain and other symptoms appear, drug therapy becomes the main treatment, including oral non-steroidal anti-inflammatory drugs (NSAIDs) or topical analgesics. Although oral medications effectively relieve pain and improve function, they cannot ensure effective drug concentration in the knee cavity, and local topical drugs are limited by their permeability. Issues such as short action time, rapid metabolism, single efficacy, and low bioavailability are major drawbacks of drug treatments for OA. (Hu et al., 2019; Liu et al., 2020) Moreover, these drugs mainly relieve pain and symptoms without directly treating OA, failing to fundamentally alleviate or inhibit disease progression. (Mao et al., 2021).

Injections of glucocorticoids (Felson et al., 2016; Orchard et al., 2020), hyaluronic acid (HA) (Altman et al., 2015; Kang et al., 2021), and platelet-rich plasma (PRP) into the joint cavity are also widely used. (Katz, 2021) However, due to rapid fluid circulation and drug metabolism in the joint cavity, drugs do not remain long, necessitating repeated injections. This increases the risk of joint infection and reduces patient compliance. Generally, current pharmacological treatments for OA are mostly palliative. For end-stage OA patients, when joint pain cannot be effectively relieved and joint movement is significantly limited, knee replacement surgery becomes a clinically and cost-effective option. (Higashi and Barendregt, 2011; Ruiz et al., 2013) However, the perioperative and lifetime postoperative risks of joint replacement surgery must be objectively evaluated (Ferket et al., 2017). Therefore, early detection and diagnosis of OA, early interventional treatment, and delaying its progression have become the focus of current clinical research. (Little et al., 2013).

With the rapid advancement of nanotechnology, nano-drug delivery systems have been extensively developed for tumor treatment and are gradually being applied to osteoarthritis (OA). These systems can achieve slow drug release in specific areas by leveraging the unique pathophysiological background of OA and responding to specific internal and external stimuli. Through modular combination, multifunctional nanomedicine delivery systems have been constructed. (Jiang and Zhang, 2023) Initially, nanoparticle delivery systems primarily functioned as carriers, loading bioactive ingredients in specific forms and achieving slow drug release in the joint cavity through intra-articular injection to enhance therapeutic effects. As nanotechnology has advanced and our understanding of the OA joint microenvironment has deepened, a series of stimulus-responsive nanoparticle delivery systems targeting OA joint microenvironment signals have been developed. These systems can respond to specific stimuli, target specific cells or tissues, alleviate inflammation, and promote cartilage regeneration. Numerous studies have reported that OA joint cavities are weakly acidic, leading to the development of a series of pH-responsive nanoparticle delivery systems for effective OA treatment. This article primarily reviews the application of pH-responsive nanoparticle delivery platforms in the treatment of osteoarthritis.

## 2 Pathological changes of pH in the joint of OA

During the progression of osteoarthritis (OA), the enhancement of the local inflammatory response produces numerous inflammatory factors and mediates tissue repair. This vigorous metabolic activity of inflammatory cells leads to a relative shortage of oxygen, inducing chondrocytes to shift towards anaerobic glycolysis. This process produces a large amount of lactic acid, gradually acidifying the joint cavity microenvironment. Additionally, inflammatory damage causes sustained degradation of articular cartilage and increased production of cartilage-degrading enzymes such as metalloproteinases. These enzymes further degrade the cartilage, releasing more degradation products that exacerbate the inflammatory response.

Inflammatory responses, cartilage degradation, and other factors collectively drive the metabolic activities within the joint cavity. Acidic substances like lactic acid are exchanged with the body primarily through synovial fluid and can be excreted. While the pH of synovial fluid in a normal joint cavity is 7.4, it gradually drops to 6.6–7.2, and can reach as low as 6.0, with the progression of OA and cartilage degradation (Treuhaft and MCCarty, 1971; Geborek et al., 1989; Andersson et al., 1999; Lee et al., 2011; Dou et al., 2020; Lombardi et al., 2022). The overall production of H^+^ in the joint cavity can be comparable to that in rheumatoid arthritis and solid tumor microenvironments. H^+^-ATPase, Na^+^-H^+^ exchange pump, and monocarboxylate transporter (MCT) are involved in the excretion of H^+^, lactic acid, and other related substances, while the exchange of anions such as Cl^−^ and HCO_3_^−^ in chondrocytes or organelles helps maintain pH stability in the joint cavity (Matsuyama et al., 2000; Webb et al., 2011; Coleman et al., 2016; Zhang et al., 2022).

On one hand, the relative hypoxia of the chondrocyte matrix aggravates the acidification of the chondrocyte microenvironment, with mitochondria-dependent apoptosis characterized by mitochondrial alkalization and cytoplasmic acidification, continuously lowering the joint microenvironment’s pH (Matsuyama et al., 2000). On the other hand, the surrounding microenvironment produces substances to buffer the H^+^ concentration, maintaining the overall stability of the joint cavity microenvironment (Vaupel et al., 1989).

## 3 Application of pH-responsive nano-drug delivery systems in osteoarthritis

In recent years, researchers have developed nanoscale, highly integrated, multifunctional hybrid delivery platforms using various physical and chemical synthesis methods. These systems have different morphologies, such as nanospheres, nanorods, and nanostars, nanoshells, which provide nanoparticles with excellent loading performance for bioactive ingredients such as drugs, peptides, and nucleic acids (Mabrouk et al., 2021). Leveraging the weakly acidic environment of the osteoarthritis (OA) joint cavity, these systems enable active and passive drug delivery with continuous slow release, extending drug residence time and increasing cellular uptake, thus achieving effective OA treatment (Zhang et al., 2022).

**FIGURE 1:**
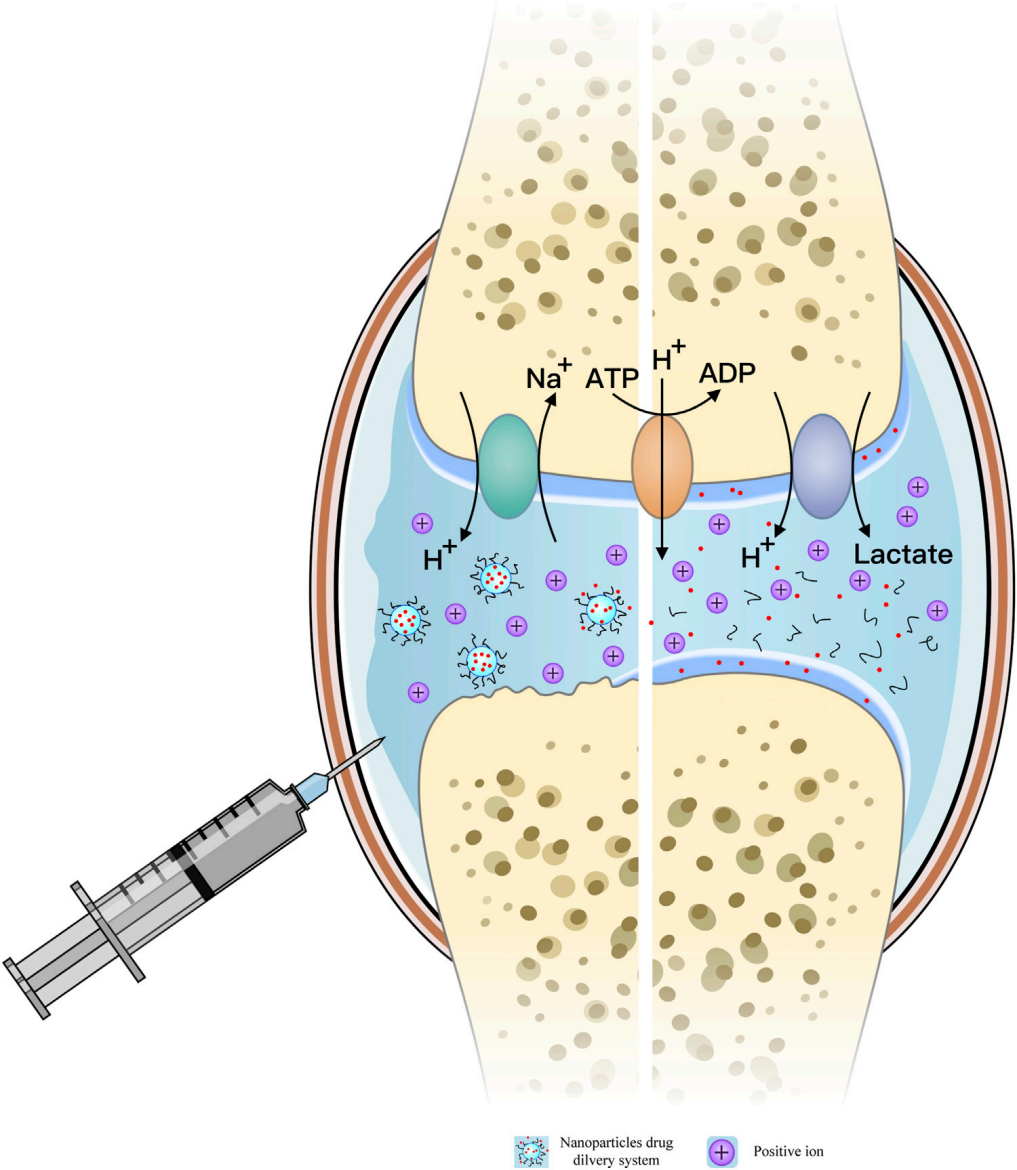
Mechanism of the weak acid microenvironment in the joint cavity of osteoarthritis (OA) patients; pH-responsive release model of nanoparticles (NPs) in the OA joint cavity.

These pH-responsive drug delivery systems respond to the low pH changes in the OA joint cavity, causing structural cracking and releasing the bioactive ingredients. Figure 1 illustrates the mechanism of the weak acidic microenvironment in OA patients’ joint cavity and the response mode of pH-responsive nanoparticles. Table 1 summarizes the material composition, bioactive ingredients, response mode and characteristics, experimental models, target sites, and results of current pH-responsive nanomaterials for OA treatment, comparing and evaluating their therapeutic effects and recent advancements.

**TABLE 1:** pH-responsive nanodrug delivery system for the treatment of osteoarthritis.

Delivery system (size)	Responsive material	Bioactive ingredient	Response mode and characteristics	Experimental model	Target site	Results	Reference
AG@MSNs-PAA (∼120 nm)	Polyacrylic acid (PAA)	Andrographolide (AG)	Acid-sensitive bond breaks	ACLT rat OA model IL-1β induced OA model	cartilage	1. Response to acidic OA environment, MSN pores are exposed 2. AG is released on demand, and inflammation is inhibited	He et al. (2021)
mPEG-Hz-b-PCL@KGN (168 ± 11 nm)	Hydrazone (Hz)	Kartogenin (KGN)	Acid-sensitive bond breaks	N/A	N/A	1. When pH = 7.4, the structure is relatively stable, pH = 5.0 hydrolysis occurs 2. However, more than 90% KGN has been released in 4 h at pH = 5.7, which is difficult to sustain	Su et al. (2022)
CSL@HMSNs-Cs (260.76 nm–290.17 nm)	HMSNs-Cs	Celastro (CSL)	Acid-sensitive bond breaks	MIA IA rat OA model IL-1β induced OA model	cartilage	1. Alleviate knee pain by reducing cytokine expression, inhibiting inflammatory response, and reducing peripheral stimulation 2. Down-regulating the expression of MMP-3 and MMP-13, promoting OA repair	Jin et al. (2020)
PCL/PEG-Nar Nanofiber Membrane (503 ± 169 nm)	PCL/PEG	Naringenin (Nar)	Acid-sensitive bond breaks	ACLT rat OA model IL-1β induced OA model	cartilage	1. Controllable release of drugs under different pH conditions 2. Protect cartilage and promote regeneration	Wang et al. (2022)
Rh-PLGA-NPs@NH4 (190.7 ± 1.2 nm)	NH_4_HCO_3_	Rhein (Rh)	Protonation	LPS induced OA model	cartilage	1. pH response releases Rh and inhibits ROS. 2. Reduce IL-1β and NO to protect cartilage	Hu et al. (2020)
MOF@HA@PCA (123.4 nm)	MOF MIL-100(Fe)	Protocatechuic acid (PCA)	Protonation	ACLT rat OA model IL-1β induced OA model	cartilage	1. Regulate drug release according to the severity of the OA, prolong the residence time of PCA in the joint cavity and slow down the OA progression	Xiong et al. (2020)
CB @ Cur@LXP	2-(Dimethylamino)ethyl methacrylate DMAEMA	Curcumin (Cur) Loxoprofen (LXP)	Protonation Dual delivery	DMM rat OA model LPS induced OA model	cartilage	1. CB has super lubrication and low wear characteristics 2. Dual drug delivery system, anti-inflammatory, ROS clearance	Zhang et al. (2023b)
MIL-101-NH@CCM-siRNA_2_ (∼200 nm)	MOF MIL-101-NH_2_	HIF-2α siRNA& Curcumin	Protonation Dual delivery	ACLT mouse OA model IL-1β induced OA model	cartilage	1. pH responds and promotes lysosome escape 2. CCM and siHIF-2α synergically inhibit hypoxia-induced cartilage dysfunction	Zhang et al. (2023a)
nano-apatite@BP (∼400 nm)	Apatite (Ca/Mg/P)	Bisphosphonates	N/A	DMM rat OA model	osteoclast	1.Inhibite overactive osteoclast bone resorption, abnormal remodeling of subchondral bone, abnormal angiogenesis and subchondral bone invasion	Geng et al. (2021)
HMPBzyme (∼210 nm)	Nanozyme HMPBzyme	N/A	pH-responsive Nanase	MIA IA rat OA model LPS-induced OA model	macrophage	1. Protect mitochondrial function and downregulate HIF-1α expression 2. Regulation of phenotypic polarization of macrophages from M1 to M2 3. Cooperate to clear ROS and relieve hypoxia	Xiong et al. (2022)
acid-activatable curcumin polymer (ACP) (∼170 nm)	Poly(beta-amino ester) (PAE)	Curcumin (Cur)	Tertiary amine protonation Fluorescence imaging	MIA IA mouse OA model LPS induced OA model	cartilage	1. The micelle remains stable under physiological conditions, performs fluorescence quenching but is easily destroyed in acidic environment to expose fluorescence 2. Inhibiting ROS production and expression of inflammatory factors TNF-α and IL-1β	Kang et al. (2020)
PLGA@HA (202.4 ± 2.3 nm)	NH_4_HCO_3_	Hyaluronic acid (HA)	Protonation NIR imaging	DMM mouse OA model	cartilage	1. Burst release at pH = 5.0 2. Combined with non-pH response NPs to achieve more durable and slow release in the later stage, revealing a new approach to OA treatment	Zerrillo et al. (2019)
MRC-PPL@PSO (121.5 ± 26.1 nm)	poly (2-ethyl-2-oxazoline)-poly (ε-caprolactone) (PEOz-PCL/PPL)	Psoralidin (PSO)	Tertiary amine Protonation NIR imaging Type II collagen targeting peptide	Papain IA rat OA model IL-1β induced OA model	cartilage	1. MMP-13 enzyme and low pH double response, drug efficient, controlled release 2. Integrated diagnosis and treatment: real-time response to OA progression and adequate treatment	Lan et al. (2020)
CMFn@HCQ (∼22 nm)	Proteins: Fn	Hydroxychloroquine (HCQ)	Protonation NIR real-time imaging Type II collagen targeting peptide	Papain IA mouse OA model IL-1β induced OA model	cartilage	1. Ferritin can produce MMP-13 enzyme in double response to low pH 2. Cleavage at low pH and recombination under neutral conditions 2. Promote cell proliferation and inhibit inflammation through ECM.	Chen et al. (2019)
pPADN-Dex (89.6 ± 9.5 nm)	pPAD	Dexamethasone (Dex)	Acid-sensitive bond breaks ROS/pH dual response Photoacoustic imaging	Papain IA rat OA model LPS induced OA model	Synovial membrane	1. ROS/pH dual-responsive, self-assembled NPs pPADN senses and eliminates excess ROS through cascade oxidation reaction 2. Delivery of glucocorticoids to inhibit synovial inflammation 3. pPADN as a photoacoustic imaging contrast agent for non-invasive examination	Zhao et al. (2021)
IA-ZIF-8@HMs (20.25 ± 0.43 μm) IA-ZIF-8 (79.57 ± 7.89 nm)	MOF: ZIF-8	Itaconic acid (IA)	Protonation Hydrogel/nanoparticles	MIA IA rat OA model IL-1β induced OA model	cartilage	1. Microfluidic technology assembles hydrogels and NPs, hydrogels delay the release of NPs 2. NPs are transported to the cellular lysozyme to release the drug	Yu et al. (2023)
AHCPA NPs@ DEX (150 ± 40 nm)	acetalated HCD materials (AHCDs)	Dexamethasone (Dex)	Protonation pH response NPs convert into hydrogels *in situ*	Collagen/LPS IA rat model	Synovium/cartilage	1. *In-situ* gelation of NPs 2. Significantly decreased the levels of TNF-α, IL-1β, IL-6, and MCP-1 3. Relieve local inflammation and cartilage erosion	Li et al. (2022)
BSA-MnO2 (BM) NPs (13.9 ± 0.4 nm) HA/PRP/BM hydrogel	Schiff base bonds	Hyaluronic acid@ Platelets-rich plasma	Protonation Hydrogel/nanoparticles	MIA IA rat OA model	cartilage	1. pH response release and ROS clearance function 2. Dual delivery of bioactive ingredients 3. Hydrogel increases lubrication and reduces wear	Zhou et al. (2022)

Note: ACLT: anterior cruciate ligament severed; DMM: medial meniscus instability; IA: articular injection; LPS: lipopolysaccharide; MIA: iodoacetate; N/A: not applicable.

pH-responsive nano-drug delivery systems offer significant advantages in OA treatment:

1. Higher drug delivery efficiency.
2. Controlled drug release rate and enhanced cellular uptake as needed.
3. High drug loading capacity, extending the administration cycle and reducing administration frequency. (Karimi et al., 2016).
4. Adjustable polymer proportions to fit the pH response range, maintaining stability at physiological pH (7.4) and gradually hydrolyzing in acidic environments, thereby mediating a cascade amplification effect for explosive drug release. (Zhou et al., 2017).

The pH response mechanism of nanomaterials primarily involves the breaking of acid-sensitive chemical bonds and the protonation of chemical groups (Liu et al., 2014; Wang et al., 2015; He et al., 2020). The former refers to the introduction of acid-sensitive chemical bonds to connect drugs and the nanoparticle structure or encapsulate drugs within the acid-sensitive nanoparticle structure. These bonds are stable at physiological pH (∼7.4) but hydrolyze in acidic environments (pH 4.5–6.5), then nanoparticles release drugs or bioactive ingredients by cracking acid-sensitive chemical bonds or reversing charges (e.g., hydrazine (Yang et al., 2010), acetate (Schlossbauer et al., 2011), imine (Yu et al., 2016) in response to pH changes in the OA microenvironment. And the latter receives or donates protons and produce pH-dependent structural and hydrophobic changes (e.g., carboxylic acid in weakly acidic and amine groups in weakly alkaline environments) to achieve drug release on demand (Wang et al., 2015; Zhao et al., 2015). Additionally, some pH-sensitive materials, such as polylysine, polyhistidine, poly(dimethyl lactamide), and poly(benzyl glutamate), can mediate the escape of nanoparticle drug delivery systems from endosomes or lysosomes and facilitate drug delivery into cells through protonation (Karimi et al., 2016; Zhou et al., 2017; He et al., 2020).

### 3.1 pH-responsive nano-drug delivery systems for OA treatment

#### 3.1.1 pH-responsive nanoparticles for OA treatment

Based on the characteristics of the weak acidic microenvironment in the joint cavity of osteoarthritis (OA), researchers have designed various nano-drug delivery systems with different compositions. These pH-responsive nanoparticles are constructed using raw materials with intrinsic pH response characteristics. For instance, Wang et al. designed a pH-responsive nanoparticles(PCL/PEG-NAR) composed of polycaprolactone and PEG naringin. This membrane was prepared by forming an ester bond between the carboxyl group of mPEG-COOH and naringin. In an acidic microenvironment, acid-promoted ester bond breaking effectively “opens” the nanofiber membrane structure, releasing naringin slowly and thereby slowing the progression of OA (Wang et al., 2022).

Hu et al. developed a polymer nanoparticle Rh-PLGA-NPs@NH4, with PLGA as the main body loaded with Rhein (Rh). In the weakly acidic joint cavity, rich in H^+^, the H^+^ penetrates the nanoparticle cladding and reacts with NH_4_HCO_3_ to produce NH_4_, CO_2_, and H_2_O. The rapid generation of a large amount of gas destroys the nanoparticle cladding, achieving explosive drug release (Hu et al., 2020). Additionally, the precursor material of nanoparticles can also be derived from organisms, offering excellent biocompatibility. Xiong et al. designed a pH-responsive biodegradable hollow structure manganese Prussian blue nanosystems (HMPBzymes). By inducing the phenotypic transformation of macrophages from M1 to M2, this system prevents and reverses the pathological progression of OA. The selection of a mesoporous structure provides a larger surface area, increases the contact area between catalyst and reactant, improves catalytic efficiency, and enhances the drug loading rate (Xiong et al., 2022).

#### 3.1.2 pH/ROS dual responsive nanoparticles for OA treatment

With the deepening of research on the pathology, molecular biology, and pharmacology of osteoarthritis(OA) and the rapid development of nanomaterials, the limitations of nano-drug delivery systems with single stimulus-response, single target, and single drug delivery have become increasingly obvious, including low target specificity, misdelivery in non-disease areas, and potential physical and chemical damage to healthy tissues caused by other stimuli. Consequently, the multi-stimulus-response model combined with a multi-target, multi-drug delivery system has emerged as a new direction for OA treatment.

Based on this approach, Zhao et al. synthesized a pegylated, phenylboric acid-modified levodopa pro-antioxidant (pPAD) that can self-assemble into nanoparticles (pPADN) for specific loading and dual-response delivery of dexamethasone (Dex) (Zhao et al., 2021). When exposed to reactive oxygen species (ROS), pPADN transforms into the active form of levodopa, exerting ROS clearance and antioxidant effects. Simultaneously, the acidic microenvironment in the joint cavity and the structural modification of pPADN promote the specific release of Dex. Multiple stimulus-response modes facilitate specific responses to different environmental changes according to the disease’s pathophysiological characteristics, reducing the incidence of erroneous drug release and incorrect targeting.

### 3.2 pH responsive nanoparticles as theranostic systems for OA treatment

Due to the lack of appropriate monitoring indicators to reflect the response process of nanoparticles *in vivo*, and the inability to accurately observe their operation path *in vivo*, the diagnosis of osteoarthritis (OA), evaluation of therapeutic effects, and tracing of therapeutic drugs remain challenging. An integrated diagnosis and treatment system is essential for achieving these functions and facilitating the efficient treatment of OA.

Kang et al. designed a self-assembled nanomicelle coated with curcumin (ACP), using poly(β-amino ester) as the main component, to achieve a rapid response in an acidic environment, release curcumin, and generate fluorescence signals at the inflammation site (Kang et al., 2020). ACP micelles were injected into the joint cavity of osteoarthritis induced by iodoacetate (MIA), and the inflammatory response was observed through fluorescence imaging. No recognizable fluorescence signal was observed in the normal joint cavity, but a significantly enhanced fluorescence signal was seen at the inflammatory site. This indicates that ACP maintains a stable micelle structure and achieves fluorescence quenching under normal physiological conditions, but under acidic conditions, the structure breaks down to expose fluorescence.

Also, Zerrillo et al. designed a polylactic-co-glycolic acid copolymer nanoparticle (PLGA NPs) coated with near-infrared dye (NIR) to track the release and uptake path of NPs in the joint lumen through optical imaging and evaluate its therapeutic effect on cartilage (Zerrillo et al., 2019). Lan et al. used a nanomicelle (MRC-PPL@PSO) as a response probe loaded with fluorescent dye cy5.5 to observe the drug targeting effect *in vivo* in real-time and continuously release the drug(Lan et al., 2020). Chen et al. designed a novel chondron-targeting, MMP-13/pH-responsive ferritin nanocage (CMFn), which contains cy5.5 and uses a quenching agent (BHQ-3) to “mask” its fluorescence signal. As OA progresses, MMP-13 enzyme activity is highly expressed, and CMFn responds to the overexpressed MMP-13 level in the OA microenvironment. It can intelligently “turn on” the fluorescence signal and induce drug release, serving both OA imaging and therapeutic functions. The detected fluorescence intensity reflects the severity of OA, enabling precise staging of the disease and ensuring timely treatment (Chen et al., 2019).The self-assembled nanoparticle (pPADN) synthesized by Zhao et al. converts into melanin-like compounds in an acidic microenvironment to activate near-infrared (NIR) photoacoustic (PA) signals, tracking the development of OA. This approach can identify inflammatory areas and monitor treatment outcomes, opening a new avenue for integrating non-invasive examination, diagnosis, and treatment (Zhao et al., 2021).

Numerous studies have confirmed the feasibility and controllability of fluorescent dyes to reflect the real-time response process of nanomaterials, promoting the combined application of various fluorescent dyes and nanomaterials. In the future, the properties of fluorescent dyes may be further utilized to reveal more pathophysiological changes in the development of osteoarthritis.

### 3.3 pH-responsive nanoparticle/hydrogel composite for OA treatment

Although pH-responsive nanodrug delivery systems have significantly improved drug delivery efficiency in the joint cavity, the pathological changes in the osteoarthritis (OA) joint microenvironment and the frequent exchange of joint fluid make it challenging for drugs to remain in the joint for extended periods. Researchers have developed several specialized material-modified nanomedical drug delivery systems targeting damaged cartilage(Chen et al., 2019; Lan et al., 2020), synovium (Nygaard and Firestein, 2020), or subchondral bone (Zhen and Cao, 2014; Yang et al., 2018) to achieve prolonged retention by anchoring to cells or the extracellular matrix, thereby extending the duration of drug action. However, targeting and lubrication capabilities still need improvement, as adequate lubrication of the articular cartilage surface is fundamental to pain relief (Clarke, 2021; Li et al., 2021). Therefore, the combination of controlled drug release and enhanced lubrication ability has been recognized as a crucial direction to further improve the efficacy of drug therapy. Researchers have introduced hydrogel systems to construct nanoparticle-hydrogel composite systems. The colloidal matrix of the hydrogel hinders the premature release of nanoparticles, allowing them to be released in response to the microenvironment.

For example, Han et al. designed a hydrogel microsphere loaded with IA-ZIF-8 (IA-ZIF-8@HMs), combining a hydrogel matrix with functional nanoparticles to impart IA-ZIF-8 with effective pH response characteristics. The hydrogel matrix further facilitates the slow release of the nanoparticles, creating a secondary structure drug delivery platform with pH response capabilities (Yu et al., 2023). Li et al. developed a pH-responsive, *in situ* gelatinized nanoparticle (AHCPA NPs) system. Under acidic conditions, the nanoparticle structure decomposes in response to pH changes, releasing various hydrophilic polyvalent host molecules. Enhanced host-guest interactions and the spontaneous assembly of polyvalent guests form an *in situ* hydrogel within the joint cavity. This hydrogel acts as a lubricant to reduce local wear and provides sustained release of dexamethasone (DEX) to minimize inflammation (Li et al., 2022). Additionally, Zhou et al. designed a dual delivery system using a hyaluronic acid (HA) cross-linked platelet-rich plasma (PRP) hydrogel network carrying bovine serum albumin-manganese dioxide nanosheets (BSA-MnO_2_). The HA/PRP/BM hydrogel, utilizing traditional raw materials like hyaluronic acid (HA), serves as a lubricant and delivery carrier. The manganese dioxide (MnO_2_) nanosheets achieve pH response and ROS clearance, triggering the release of multiple payloads under acidic conditions, including the total protein of PRP and the representative growth factor transforming growth factor β1 (TGF-β1). (Zhou et al., 2022).

We have deleted some content of sections 5 and 6 and integrated them into section 7(5) for a brief explanation. We have also revised the relevant content in section 7(5) to make it more academically styled and enhance the readability of the article.

## 4 Conclusions and prospects

Based on the information summarized in Table 1, we understand that pH-responsive nanoparticles can successfully respond to the weakly acidic microenvironment within the osteoarthritic joint cavity. However, challenges such as burst release and insufficient sustained release remain. By integrating additional response mechanisms, we can achieve more controllable drug release. Furthermore, incorporating hydrogel systems not only facilitates nanoparticle release but also provides lubrication.

Some nanoparticles, either alone or loaded with fluorescent dyes, can serve imaging functions to monitor the progression of osteoarthritis in real-time. Despite extensive research on nanoparticles for osteoarthritis (OA) treatment, current nanoparticle delivery systems have many limitations. Many functionalities of nanoparticles remain unexplored, and several pathophysiological changes in osteoarthritis are still not thoroughly elucidated.

On the one hand, we need to consider the safety of nanoparticle metabolism in the body. Protonation-induced escape from the endoplasmic reticulum and lysosomes can lead to the leakage of hydrolases into the cytoplasm, resulting in autophagy and cell death (Mura et al., 2013). Additionally, pH-responsive nanomaterials based on acid-sensitive chemical bond response mechanisms can be partially cytotoxic due to the presence of ketone or aldehyde functional groups and cationic polymer residues (Sonawane et al., 2017). Some nanoparticles can interact closely with the immune system, potentially inducing hypersensitivity reactions, and trigger the overproduction of reactive oxygen species (ROS) in the body (Mitragotri and Lahann, 2009; Feng et al., 2019).

On the other hand, the preparation of pH-responsive drug delivery systems should comprehensively consider the characteristics of the material, the pathophysiological characteristics of OA, the composition and physical and chemical properties of the drug, and the optimal therapeutic dose. Common targets for pH-responsive nanomaterials in osteoarthritis (OA) treatment, such as cartilage, synovium, and subchondral bone, are insufficient. In addition to exploring drug release at the target, the impact of disease-specific anatomical and physiological barriers (e.g., extracellular matrix (ECM)) on the bioavailability of nanomaterials must be considered (Lavrador et al., 2018; Majumder and Minko, 2021). It is essential to deepen our understanding of OA at the molecular level. Additionally, and promote the development of new materials to discover more target-specific and biocompatible drug carriers. Thus, it opens up a new and effective way for disease treatment (Mura et al., 2013; Lavrador et al., 2018; Raza et al., 2019).

At present, the application of pH-responsive drug delivery systems in the treatment of osteoarthritis (OA) primarily focuses on the low pH characteristic, the designed nanomaterials crack under acidic conditions, causing the release of bioactive components. Some studies have further utilized material characteristics to assemble multifunctional modules, achieving dual and multiple targeting capabilities, such as type II collagen targeting peptide (Lan et al., 2020) and specific gene knockout sequences (siHIF-2α) (Zhang ZJ. et al., 2023) Additionally, fluorescence can be loaded and specifically regulated to “turn on and off,” providing imaging capabilities and real-time visualization of the progression and severity of osteoarthritis (Chen et al., 2019). Although there are changes in pH, reactive oxygen species (ROS), and matrix metalloproteinases (MMPs) in damage tissues(Feng and Chen, 2018; Dou et al., 2020), material characteristics or bioactive ingredients, primarily target cartilage to achieve therapeutic effects for osteoarthritis (OA) by alleviating or improving cartilage lesions. There are still few studies focusing on the synovium and subchondral bone. It is impossible to achieve accurate drug release in a complex microenvironment with multiple signals by relying on a single stimulus mode. This limitation increases the “false positive” rate and the risk of non-targeted delivery (Municoy et al., 2020).

By equipping materials with multiple response capabilities, we can construct multi-stage reaction systems for drug delivery, and combine imaging and treatment through the use of fluorescent dyes. For example, pH and ultrasound (US) response materials can be used to improve both drug release and cellular uptake. Magnetic and US response materials have heating effects, while magnetic response materials also possess unique magnetic permeability properties. The unique advantages of different response systems become the key to optimizing carrier performance and achieving multiple response capabilities. For example, MMP-13/pH-responsive ferritin nanocages are modified to respond to the overexpression of MMP-13 and pH changes in OA joint cavities. These nanocages reflect the progression of osteoarthritis in real-time by intelligently “turning on” fluorescence imaging and releasing anti-inflammatory drugs (Chen et al., 2019). More importantly, the multistage response capability of the composite system allows the carriers to be altered through two or more stimuli, enabling batch and on-demand release of drugs (Costa Lima and Reis, 2015). However, given the uncertainty of interactions between different materials and their interference, it is crucial to objectively evaluate the overall therapeutic effect of dual and multiple response systems. Blindly combining different stimuli could potentially have adverse effects.

In designing new materials, we should consider not only the specificity of target selection but also the accuracy of targeting. For osteoarthritis (OA), the complexity of physiological variables such as disease status, blood flow, and tissue structure must also be considered (Muro, 2012; Noble et al., 2014). Currently, many advanced dual and multiple response systems are concentrated in cancer research, But, due to the common pathological characteristics of tumors and inflammation, these advancements are still of great guiding significance for developing drug delivery systems for OA treatment.

In the future, the development of nanoparticle delivery systems should aim to build multi-drug delivery platforms to overcome the limitations of single-drug delivery, which cannot achieve multi-functional therapeutic effects such as anti-inflammatory, antioxidant, and cartilage repair functions. Although some studies have successfully implemented dual bioactive component delivery systems for the treatment of osteoarthritis (OA), such as the pH-responsive nanoparticle dual drug delivery system based on circular brush zwitterionic polymer that successfully loaded hydrophobic curcumin and hydrophilic loxoprofen sodium (CB@Cur@LXP), the circular brush also has joint lubrication and ROS removal properties (Zhang M. et al., 2023). Additionally, a chondro-targeted dual drug delivery nano platform (RB@MPMW) was developed to conjugate Type II collagen targeting peptide (WYRGRL) and load rapamycin (Rap) and bilirubin (Br) with NIR laser irradiation to produce a photothermal effect for drug release (Xue et al., 2021). However, current dual-drug delivery systems have high requirements on the properties of the drugs themselves (e.g., polarity, hydrophobicity) and cannot achieve broad multi-drug loading and high load efficiency.

In conclusion, to achieve more accurate and efficient treatment of OA with pH-responsive targeted nanomaterials, and further promote the research and development of new materials to find more target-specific and biocompatible drug carriers. We must optimize target selection, targeting capability combinations, and material and drug combinations. Actively exploring a variety of signals of pathophysiological changes in OA patients, and not limiting our focus to targeting cartilage, will be crucial. The development direction for treating various diseases, including OA, will be nanoparticle drug delivery systems with multi-stimulus response modes, multi-functional targeting, and multi-drug delivery capabilities.
